# The Interplay of Protein Aggregation, Genetics, and Oxidative Stress in Alzheimer’s Disease: Role for Natural Antioxidants and Immunotherapeutics

**DOI:** 10.3390/antiox13070862

**Published:** 2024-07-18

**Authors:** Jawad Ali, Kyonghwan Choe, Jun Sung Park, Hyun Young Park, Heeyoung Kang, Tae Ju Park, Myeong Ok Kim

**Affiliations:** 1 Division of Life Science and Applied Life Science (BK21 FOUR), College of Natural Sciences, Gyeongsang National University, Jinju 52828, Republic of Korea; jawadali666@gnu.ac.kr (J.A.); k.choe@gnu.ac.kr (K.C.); jsp@gnu.ac.kr (J.S.P.); 2 Department of Psychiatry and Neuropsychology, School for Mental Health and Neuroscience (MHeNs), Maastricht University, 6229 ER Maastricht, The Netherlands; hailey.park@maastrichtuniversity.nl; 3 Department of Pediatrics, Maastricht University Medical Center (MUMC+), 6202 AZ Maastricht, The Netherlands; 4 Department of Neurology, Gyeongsang National University Hospital & College of Medicine, Gyeongsang National University, Jinju 52727, Republic of Korea; heeyoung@gnu.ac.kr; 5 Haemato-Oncology/Systems Medicine Group, Paul O’Gorman Leukaemia Research Centre, Institute of Cancer Sciences, College of Medical, Veterinary & Life Sciences (MVLS), University of Glasgow, Glasgow G12 0ZD, UK; 6 Alz-Dementia Korea Co., Jinju 52828, Republic of Korea

**Keywords:** Alzheimer’s disease, amyloid beta (Aβ), oxidative stress, natural antioxidants, immunotherapy

## Abstract

Alzheimer’s disease (AD) is a progressive neurodegenerative disorder that comprises amyloid-beta protein (Aβ) as a main component of neuritic plaques. Its deposition is considered a trigger for AD pathogenesis, progression, and the clinical symptoms of cognitive impairment. Some distinct pathological features of AD include phosphorylation of tau protein, oxidative stress, and mitochondrial dysfunction. These pathological consequences tend to produce reactive oxygen species (ROS), resulting in the dysregulation of various signaling pathways of neuroinflammation and neurodegeneration. The relationship between the Aβ cascade and oxidative stress in AD pathogenesis is like a “chicken and egg” story, with the etiology of the disease regarding these two factors remaining a question of “which comes first.” However, in this review, we have tried our best to clarify the interconnection between these two mechanisms and to show the precise cause-and-effect relationship. Based on the above hallmarks of AD, several therapeutic strategies using natural antioxidants, monoclonal antibodies, and vaccines are employed as anti-Aβ therapy to decrease ROS, Aβ burden, chronic neuroinflammation, and synaptic failure. These natural antioxidants and immunotherapeutics have demonstrated significant neuroprotective effects and symptomatic relief in various in vitro and in vivo models, as well as in clinical trials for AD. However, none of them have received final approval to enter the drug market for mitigating AD. In this review, we extensively elaborate on the pitfalls, assurances, and important crosstalk between oxidative stress and Aβ concerning current anti-Aβ therapy. Additionally, we discuss future strategies for the development of more Aβ-targeted approaches and the optimization of AD treatment and mitigation.

## 1. Introduction

Alzheimer’s disease (AD) is the most common cause of neurodegenerative disorder, leading to a gradual decline in cognitive and memory function. AD is characterized by the deposition of amyloid plaques (Aβ) in the extracellular space and neurofibrillary tangles (NFTs) in the intracellular compartment of the brain [1]. Both Aβ plaques and NFT aggregation are major hallmarks of AD pathology that interfere with neuronal communication at the synapses; as well as occlude essential nutrient transport within neuronal tissue [2]. In the amyloidogenic pathway, Aβ is synthesized from amyloid precursor protein (APP) by the consecutive cleavage of β-secretase and γ-secretase, while in the non-amyloidogenic pathway, α-secretase is the predominant enzyme for Aβ synthesis [3]. Along with this, abnormal fiber bundles formed through tau hyperphosphorylation by various kinases are also present in the AD brain [4]. Early onset of AD is connected to three rare forms of autosomal-dominant gene mutation that encode for amyloid precursor protein (*APP*), presenilin 1 (*PSEN1*), and presenilin 2 (*PSEN2*), while apolipoprotein E (*APOE*) genes are associated with late onset of AD. These gene mutations affect the production of the Aβ peptide, a key component of senile plaques [5,6]. In addition to APOE, other risk variants in genes for late-onset AD, including *ADAM10*, *ADAMTS1*, *MAPT*, *GRN*, *ARSA*, and *CSF1R*, are involved in APP and tau metabolism [7].

The clearance pathways for Aβ and tau follow various mechanisms, including lysosomal degradation, ubiquitination and degradation via the proteasome, microglial engulfment, and transport through interstitial fluid (ISF), the blood–brain barrier (BBB), and cerebrospinal fluid (CSF) [8]. Two transmembrane receptors on endothelial cells, lipoprotein receptor-related protein (LRP) and receptor for advanced glycation end products (RAGE), are responsible for the transport of soluble Aβ from the brain into the blood and vice versa [9]. In addition, the ATP-binding P-glycoprotein transporter (P-gp) transports soluble Aβ across neuronal endothelial cells into the bloodstream; however, it has also been discovered that gp330/megalin transports circulating Aβ from plasma back into the brain via apolipoprotein J (ApoJ) [10]. In AD brains, it has been confirmed that expression of LRP efflux is reduced, whereas blood influx of the RAGE transporter is upregulated and sustained [11]. Enzymatic degradation of Aβ occurs via different peptidases, specifically zinc metalloendopeptidase family members, neprilysin, and insulin-degrading enzyme (IDE), while other members of the matrix metalloproteinase (MMPs) family include angiotensin-converting enzyme (ACE) and endothelin-converting enzyme (ECE) [12]. In aging and AD brains as well, some epidemiological studies have shown decreased levels and activities of neprilysin, IDE, and ACE [13].

In contrast to Aβ, abnormally phosphorylated NFTs appear to contribute, with or without concomitant aggregation of amyloid plaques, not only affecting axonal transport and synapses but also disturbing the stress response in cells [14]. NFTs affect characteristic laminar and regional distribution, including the transentorhinal region, the pyramidal neurons of the hippocampus, the amygdala, the parahippocampus, higher multimodal areas of the neocortex, and subcortical regions of the brain [15]. Preclinical investigations of both Aβ and NFTs using neuroimaging studies, such as positron emission tomography (PET), aim to determine Aβ occurrence in the tau-classified Braak staging regions and explore their relationship with cognitive impairments [16]. Although many recent fundamental treatment strategies for AD currently exist, they are either symptomatic, disease-modifying, or in active phase 3 disease-modifying drug clinical trials. In addition, natural antioxidants, including herbals, phytopharmaceuticals, medicinal products, and nutraceuticals, are also becoming promising candidates for the management of AD [17].

## 2. Structural and Pathological Aggregation Cascade of Aβ

**Figure 1:**
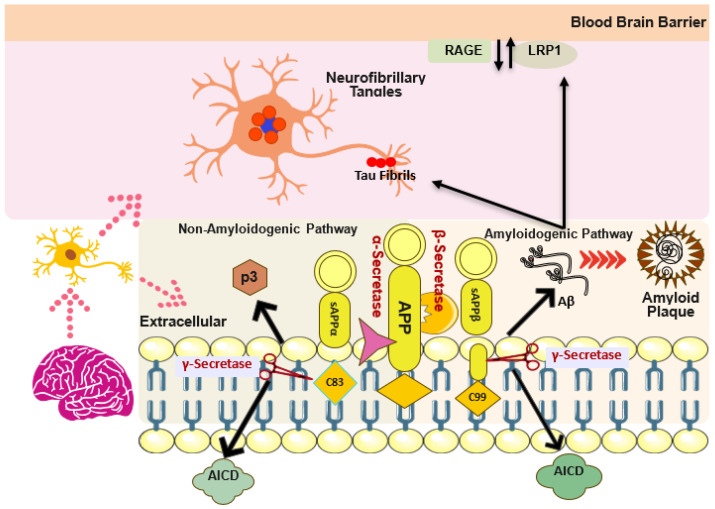
Amyloid-beta cascade comprising non-amyloidogenic and amyloidogenic processing of APP via β-secretase, α-secretase, and γ-secretase. Similarly, phosphorylation of tau via Aβ to form NFTs, as well as Aβ transport via RAGE/LRP1 receptor.

Amyloid precursor protein (APP) is the best-known integral membrane protein found in neuronal synapses, playing a key role in the production and pathological aggregation of Aβ [18]. APP crosses the lipid bilayer of the cell membrane and consists of a large extracellular glycosylated N-terminus and a shorter cytoplasmic C-terminus [19]. Human APP proteolytic cleavage occurs via two alternative pathways: amyloidogenic and non-amyloidogenic, resulting in a peptide of 37 to 49 amino acids known as Aβ, which is a primary component of amyloid plaques in the brains of AD patients [20]. In the amyloidogenic pathway, APP degradation is carried out by the consecutive action of β-secretase (BACE-1) at the Asp site, resulting in the synthesis of secreted N-terminal sAPPβ and γ-secretase, which produces membrane-bound C-terminal fragments (C99 or CTFβ) (Figure 1). CTFβ is further degraded by γ-secretase at multiple sites, producing fragments of 43, 45, 46, 49, and 51 amino acid residues. These fragments ultimately lead to the formation of extracellular Aβ peptides, specifically Aβ40 and Aβ42, along with CTFγ [21]. In contrast, the non-amyloidogenic pathway utilizes α-secretase, which cleaves APP at the Leu site. This process produces the secreted N-terminal sAPPα and membrane-bound C-terminal fragments (C83 or CTFα). CTFα is further degraded by γ-secretase into the extracellular P3 peptide and CTFγ [22]. All these Aβ monomers aggregate into various structures, including oligomers, protofibrils, and mature amyloid fibrils, which are insoluble forms that can be further aggregated into amyloid plaques, most significantly found in the neocortex of AD patients [23].

## 3. Structural and Pathological Aggregation, Phosphorylation of Tau Protein

Microtubule-associated tau protein is produced by the microtubule-associated protein tau (*MAPT*) gene and is essential for the stabilization of the cytoskeleton of neurons and their cell division [24]. The alternative splicing of the *MAPT* gene results in six distinct isoforms of tau, which accumulate to form protofibrils that eventually convert into fibrils exhibiting a paired helical filamentous structure (PHFs) [25]. Normally, the phosphorylation of tau is a physiological activity essential for axonal transport, microtubule integrity, and neuronal development [26]. Abnormal tau phosphorylation occurs at sites including threonine and serine (phospho-threonine 231), (pSer202/Thr205), (pSer396/pSer404), and (pThr212/pSer214). These phosphorylation events lead to tau aggregation in the form of pretangles, the paperclip conformation (MC1), and ultimately neurofibrillary tangles or ghost tangles in the later stages of AD. This process provides evidence of the destabilization of the physiological balance of tau phosphorylation [25]. In addition, NFTs contribute to neuropathological changes by forming pretangles or neuropil threads that create lesions. These lesions propagate and distribute throughout various regions of the AD brain, following patterns first described by Braak and Braak [16].

## 4. Aβ and Tau Interplay

Previous data support that Aβ not only accelerates tau phosphorylation but also interferes with tau oligomerization, thus showing Aβ as a “trigger” and tau as a “bullet” in driving AD [27]. Multiple protein kinases targeting serine or threonine, including cyclin-dependent kinase 5 (CDK-5), glycogen synthase kinase-3β (GSK-3β), mitogen-activated protein kinases (MAPKs), and tyrosine kinases such as Src family kinases (e.g., FYN), contribute to the hyperphosphorylation of tau under pathological conditions [28]. The GSK-3β and CDK-5 pathways are intricately linked to Aβ aggregation, leading to tau hyperphosphorylation through the increased activity of both GSK-3β and CDK-5, ultimately resulting in Aβ-induced tauopathy [29]. Similarly, Aβ-induced tau hyperphosphorylation involves several MAPKs (ERK1/2, SAPK/JNK, and p38), all of which utilize tyrosine phosphorylation, leading to neurofibrillary degeneration in AD [30]. In addition, Aβ initiates tau aggregation via the caspase-3 (CASP3) enzyme in the C-terminus of Asp421, yielding an N-terminal product. This truncated tau more rapidly assembles into small neurotoxic filaments than the actual tau length [31]. Furthermore, Aβ produces 17-kDa tau fragments in the hippocampus through calpain-1 activity. These cleaved tau fragments tend to aggregate, misfold, and propagate not only to neuronal compartments but also bind to glial cells to induce neuroinflammation and neurodegeneration [32]. Similarly, at the cellular and biochemical levels, Aβ oligomers form complexes with cellular prion protein (PrPC), activating various downstream kinases, including Pyk2 (Ptk2b) and Fyn, which interact with GSK-3β and lead to tau phosphorylation. There is evidence of a pathological interaction between Aβ and PrP concerning tau phosphorylation, neurotoxic signaling, and nerve cell death in APP23xTAU58 mice and the human AD brain [33].

## 5. Early and Late-Onset of AD and Genetics

Age is a well-known biological risk factor, particularly between 40 and 65 years, which is classified as early-onset AD (EOAD). Amyloid precursor protein (*APP*), presenilin 1 (*PSEN1*), and presenilin 2 (*PSEN2*) are autosomal dominant genes, and mutations in all three are associated with EOAD. Along with typical clinical symptoms of cognitive impairment, some atypical symptoms of visual acuity loss, dyscalculia, apraxia, and aphasia have also been more frequently reported in EOAD [34]. According to previous reports, the *APP* gene undergoes 52 pathogenic mutations, *PSEN1* is the most mutated gene with 215 mutations, and *PSEN2* has 31 genetic mutations (*PSEN1* > *APP* > *PSEN2*). The mutational spectrum of these genes includes missense mutations, duplications, and small insertions or deletions of amino acids, all of which contribute to increased susceptibility to Alzheimer’s disease [35]. In late-onset AD (LOAD), typical clinical symptoms develop after 65 years of age with a complex heterogeneous etiology and a heritability rate of 70 to 80%. Apolipoprotein E (*APOE*) has three isoforms (*APOE2*, *APOE3*, and *APOE4*), which impact Aβ aggregation and clearance in distinct ways. Among these, the ε4 allelic form of the APOE4 gene is the major genetic risk variant for LOAD, as identified via genome-wide association studies (GWAS) in patient/control cohort studies [36]. In addition, whole genome sequencing has identified other genetic risk variants involved in APP and tau metabolism (such as *ADAM10*, *ADAMTS1*, and *MAPT*) or genes associated with dementia (e.g., *CSF1R*, *GRN*, and *ARSA*). This provides evidence of their involvement in lipid metabolism, endocytosis, immune response, and synaptic function in the development of LOAD [7].

## 6. Aβ and Tau Interaction with Synapse

Aβ and tau may play a key role in healthy synaptic physiology in the form of synaptic plasticity, microtubular stability, and axonal transport. The enzymes (APP, beta, and gamma secretases) required for Aβ generation are also present in the synapse. Additionally, tau’s regulation of glutamate receptors (NMDAR) through the activation of Fyn kinases provides substantial evidence of normal synaptic physiology, specifically supporting memory and cognition [37]. On the other hand, abnormal aggregation of Aβ and tau neurofibrillary tangles in the brain is associated with synaptic loss and axonal degeneration, respectively. However, the molecular mechanisms underlying synaptic dysfunction via Aβ and tau are not yet fully understood, although several possible pathways have been elucidated through multiple research studies [38]. One study found evidence that Aβ increases Ca^2+^ influx into the dendritic spines and activates downstream calcineurin, as observed around amyloid plaques in vivo in AD transgenic mice. Both changes in calcium concentration and calcineurin activation by Aβ disturb normal synaptic plasticity [39]. Another study provides evidence that Aβ, along with non-apoptotic caspase, disturbs AMPA and NMDA receptor activity via the calcium-calcineurin-mediated pathway. This disturbance leads to impaired glutamate reuptake, suppression of long-term potentiation (LTP), enhancement of long-term depression (LTD), synaptic loss, and ultimately neuronal cell death [40]. Similarly, irregular aggregation of Aβ directly interacts with pre-synaptic proteins involved in synaptic vesicle fusion, exocytosis, recycling, and recovery for neurotransmitter release from the pre-synaptic terminal. This interaction targets post-synaptic SNARE phosphorylation (Syntaxin-3, Syntaxin-4, SNAP-23, SNAP-25) and Synaptobrevin-2 (VAMP2) in dendrites or spines [41]. In parallel to Aβ, the trans-synaptic distribution of oligomeric tau is found in higher concentrations at the presynapse and gradually decreases at the postsynapse, exhibiting a neurotoxic nature. Amyloid beta plaques are extracellular lesions, while neurofibrillary tangles (ghost tangles) are intracellular lesions. Both promote synaptic alterations and loss in the early stages of AD-affected brain compartments and best correspond to cognitive impairment [42].

## 7. Oxidative Stress and Different Pathways in AD

Oxidative stress is defined as alterations in the antioxidant mechanism involving the production and accumulation of reactive oxygen species (ROS), such as superoxide (O^2•−^), hydrogen peroxide (H_2_O_2_), peroxynitrite (ONOO^−^), hydroxyl radicals (HO^•^), and reactive nitrogen species (RNS). These highly reactive species act as signaling molecules in various pathways of AD [43]. In normal situations, there is a balance between the production of reactive oxygen species (ROS) and reactive nitrogen species (RNS) in cells and their removal by an antioxidant defense system, which comprises various antioxidant enzymes (such as SOD, catalase, and GST). When this balance is disrupted, it results in oxidative stress. The brain is a vital organ with high energy consumption, a greater demand for oxygen, an abundance of oxidizable polyunsaturated fatty acids, and a significant susceptibility to oxidative stress [44]. Increased ROS production in neuronal cells not only leads to polypeptide breakdown through the removal of hydrogen atoms from the alpha carbon but also oxidizes various amino acids (such as Arg, Lys, and Ser) and polyunsaturated fatty acids to form protein carbonyls. These carbonylated proteins exhibit altered polarity, with changes in their secondary, tertiary, and quaternary structures; tend to aggregate as cytoplasmic inclusions within neurons, and are less prone to degradation via proteasome [45].

### 7.1. Oxidative Stress and Different Pathways in AD

#### 7.1.1. Oxidative Stress and Mitochondria Dysfunction in AD

Neuronal cells have abundant mitochondria, which serve as the main powerhouse and supply energy in the form of ATP via oxidative phosphorylation (OXPHOS), essential for neuronal transmission and synaptic plasticity. Mitochondrial dysfunction is a major factor in oxidative stress due to unavoidable electron leakage during transfer via the electron transport chain (ETC), which is converted to superoxide anions. This process leads to the production of 90% of endogenous ROS and contributes to oxidative imbalance, an early feature of AD brains [46]. Several studies have demonstrated that various oxidative metabolic enzymes, such as α-ketoglutarate dehydrogenase, pyruvate dehydrogenase, ATP synthase, and cytochrome c oxidase, in the mitochondrial ETC exhibit significantly decreased activity and impaired energy supply in the AD brain [47,48]. In addition, mitochondrial DNA mutations, including 5 kb deletions, the absence of protective histones, and oxidized nucleosides, make mtDNA more vulnerable to ROS in AD patients. These mutations commonly affect mtDNA at transcriptional and replication levels, leading to the generation of many altered mitochondrial proteins [49]. Moreover, mitochondrial dynamics, including structural damage in the form of broken cristae, partial or complete internal structure loss, and a slight increase in size, all contribute to the disturbance of the mitochondrial intact structure and the proper functioning of the electrochemical gradient. These changes are observed in biopsied AD brain tissue, as displayed via electron microscopy [50]. Similarly, mitochondria undergo structural and functional abnormalities due to an imbalance in GTPase genes that regulate their constant division and fusion. These genes facilitate mitochondrial movement from the cell body to nerve terminals and synapses to meet energy demands and include dynamin-related protein 1 (Drp1), fission 1 (Fis1), and the fusion proteins mitofusin 1 and 2 (Mfn1, Mfn2), as well as optic atrophy protein 1 (Opa1). Multiple studies provide sufficient evidence of significant alterations in the expression of these fission and fusion genes in AD brains [51,52].

#### 7.1.2. Oxidative Stress and Aβ Interplay

Oxidative stress has been proposed as a key trigger in AD since the preliminary stage of mild cognitive dysfunction. There is cross-talk between ROS-induced oxidative stress and AD cascades through different mechanisms, including irregular proteostasis of both Aβ and tau filaments, mitochondrial dysfunction, lipid peroxidation, impaired antioxidant defense systems, neuroinflammation, and, last but not least, DNA damage [53]. Both oligomeric and fibrillar forms of Aβ are recognized as neurotoxic in human neurons, particularly through the induction of ROS, which causes abnormal function of the NMDA receptor and ultimately leads to cell death via activation of the NADPH oxidase pathway and superoxide anions. Additionally, there is sufficient evidence of metal ions (Cu^2+^, Zn^2+^, and Fe^2+^) interacting with Aβ, leading to enhanced oxidative activity in the form of free radicals and hydroxyl ions, ultimately resulting in oxidative stress [54]. Inversely, similar to the chicken-and-egg, which comes first, ROS-induced oxidative stress affects Aβ production at the genetic, transcriptional, and translational stages by disrupting various signaling pathways [55].

#### 7.1.3. Oxidative Stress and Tau Interplay

Increased levels of oxidative stress play a prominent role in the pathophysiology of numerous neurodegenerative disorders, including tauopathies. There is evidence that the accumulation of ROS can lead to tau hyperphosphorylation and aggregation [56]. Cross-talk between ROS and tauopathy shows a close association in several clinical patients as well as in animal models. For example, patients with Pick’s disease (frontotemporal dementia) and corticobasal degeneration (CBD), as well as transgenic mouse models of frontotemporal lobar degeneration (FTLD-tau), exhibit elevated levels of various oxidative markers such as malondialdehyde (MDA), 4-hydroxynonenal (4-HNE), SOD1, SOD2, and heme oxygenase-1 (HO-1) [57]. Similarly, irregular accumulation of tau leads to ROS generation by affecting mitochondrial function, resulting in reduced activity of complex I and complex V, ATP depletion, impaired membrane potential, fragmentation, and finally defective mitochondria in AD [58]. Some evidence has shown that truncated tau with N-terminal (NH₂-26–44) is found in the mitochondrial membrane, causing an imbalance in mitochondrial membrane potential (ΔΨm) and ATP synthesis, along with Aβ, in the neuronal tissue culture. To support this, another study reported that truncated tau at Asp421, generated via caspase-3 and Aβ, halts mitochondrial function and may be a primary inducer of oxidative stress in AD [59]. On the other hand, post-translational modifications (PTMs) in tau, including oxidation, nitration, and glycosylation, can all result from elevated ROS. To confirm this, several studies have shown that oxidative stress not only affects the activity of various tau kinases and phosphatases but also regulates tauopathies through different mechanisms, such as lipid peroxidation, which stimulates tau hyperphosphorylation and oligomerization [60].

#### 7.1.4. Oxidative Stress and Calcium Dyshomeostasis Interplay

**Figure 2:**
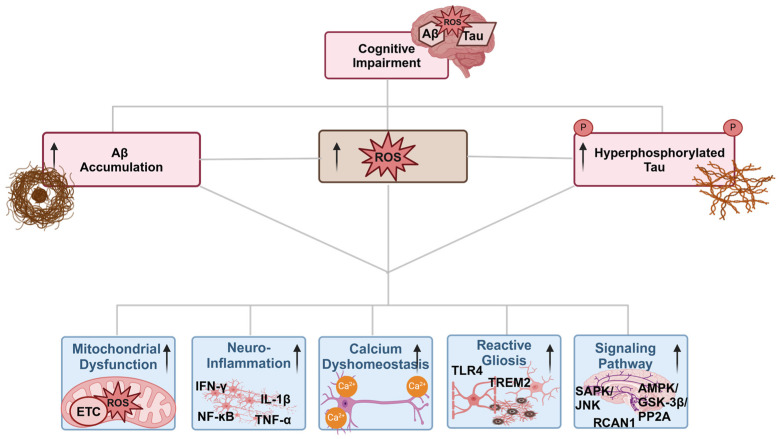
Cognitive impairment due to accumulation of Aβ plaques, phosphorylated tau protein, and ROS, along with their interplay. Aβ, ROS, and phosphorylated tau, in turn, interact in ways that involve mitochondrial dysfunction, neuroinflammation, calcium dyshomeostasis, reactive gliosis, and dysregulation of various signaling pathways in neuroinflammation-induced AD.

Aβ oligomer toxicity leads to the disruption of the cell membrane’s phospholipid bilayer, allowing an increase in cytoplasmic Ca^2+^ ion levels from the extracellular space and the generation of ROS in human neuroblastoma SH-SY5Y cells as well as rat cortical neurons [61]. The dysregulation of calcium ion influx may occur at all stages of AD and plays a prominent role in triggering synaptic failure, mitochondrial dysfunction, oxidative stress, and neuroinflammation. These alterations indicate that calcium signaling affects multiple neuronal processes, including proliferation, maturation, migration, and differentiation of progenitor neurons (Figure 2). In addition, it also includes synaptic plasticity, as it interferes with neurotransmitter release from the pre-synaptic terminal and disrupts gene expression by acting as a second messenger. Moreover, Ca^2+^ influx in neuronal cells tends to accumulate ROS in mitochondria due to the increased production of ATP needed to pump out Ca^2+^ [62]. On the other hand, the accumulation of ROS can significantly affect Ca^2+^ homeostasis both within the cell and in intracellular Ca^2+^ stores by oxidizing the signaling protein calmodulin (CaM) at multiple methionine residues. This leads to the inactivation of the cell membrane Ca^2+^-ATPase, which is essential for maintaining Ca^2+^ homeostasis [62].

#### 7.1.5. Oxidative Stress and Signaling Pathways Interplay

Alterations in gene expression and enzyme activity are mediated by cellular stress, particularly oxidative stress, which occurs through the impairment of multiple signaling pathways, including stress-activated protein kinase (SAPK/JNK), CREB/ERK, RCAN1, AMPK/GSK-3β/PP2A, and Nrf2-ARE, among others. These pathways are dysregulated due to the overproduction of ROS in the AD patient’s brain and are one of the leading causes of neuroinflammation and neurodegeneration [63]. Previous reports suggest that SAPK pathways play a significant role in various cellular processes, ranging from oxidative stress to cell death. For instance, JNK2 and JNK3 are associated with neurofibrillary tangles, while JNK1 is linked to Hirano bodies in chronic conditions like AD. Importantly, JNK/SAPK activation not only promotes amyloid deposition but also results in its nuclear localization, which is uniformly detected in many vulnerable neurons in the early stages of AD, exhibiting a pattern similar to the oxidative marker 8-OHdG. This provides strong evidence that oxidative stress is a key activator of the JNK/SAPK pathway in AD [64]. In addition, the cyclic AMP-regulated element-binding protein (CREB) plays a crucial role in regulating various neurotrophic factors and is an important signaling molecule for cognitive function and neuronal survival. Any alterations in nerve growth factor (NGF) and epidermal growth factor (EGF) due to oxidative stress can trigger a signaling cascade involving the phosphorylation of serine kinases (Ras, Raf, ERKs, and p38), ultimately activating CREB and resulting in neuronal dysfunction in rat pheochromocytoma PC12 cells [65]. Moreover, RCAN1, a regulator of calcineurin, is highly expressed in neuronal tissue and interacts with several kinases, including NF-κB-inducing kinase (NIK), TAK1, and especially GSK-3β. This interaction can lead to the hyperphosphorylation of tau, which presents the first signs of neurodegeneration. It has been reported that long-term RCAN1 gene production can lead to the upregulation of GSK-3β, which alters mitochondrial functions in several ways, such as diminished adenine nucleotide translocators (ANT), an imbalanced ATP/ADP ratio, mitochondrial permeability transition (mtPTP), and ultimately mitochondrial death, leading to oxidative stress [66].

Similarly, the AMPK/GSK-3β/PP2A pathway serves as an important cellular energy sensor that regulates ATP metabolism through mitochondrial biogenesis and ROS management. It is well known that GSK-3β activation leads to a downstream factor called protein phosphatase 2A (PP2A), which is primarily associated with mitochondrial apoptosis and the pathogenesis of AD. The same study reports that oxidative stress can directly induce hyperphosphorylation of tau by elevating the levels of both GSK-3β and PP2A in AD [63]. Last but not least, the most important transcriptional pathway in neurodegenerative diseases is the Nrf2/ARE pathway, as it regulates redox homeostasis, DNA repair, and mitochondrial autophagy. The transcription factor nuclear factor (erythroid-derived 2)-like 2 (Nrf2), a master regulator of the oxidative stress response, binds to a cis-acting element known as the antioxidant responsive element (ARE), protecting the cell from oxidative stress-induced neuronal cell death [67]. In addition, several protein kinases, such as casein kinase 2, protein kinase C (PKC), and MAPK, regulate the phosphorylation of Nrf2, facilitating its release from Keap1 and translocation to the nucleus. Nrf2 also interacts with co-activators such as CREB binding protein/p300 and chromatin remodelers, which bind to ARE protein binding sites to initiate the transcription of several antioxidant enzymes, including GST, CAT, SOD, HO-1, and NADPH-regenerating enzymes [67]. In AD, any disturbance to the Nrf2/ARE pathway can ultimately lead to irregular production or function of several kinases, promoting APP cleavage and tau oligomerization. Subsequently, the overexpression of Aβ and tau decreases Nrf2 levels and transcription of protective genes, further impairing the ETC in mitochondria and ultimately increasing oxidative stress [68]. Simultaneously, several kinases negatively regulate the Nrf2 pathway in pathological conditions like chronic inflammation and AD. Among these is GSK-3β, which is upregulated in post-synaptic fractions and the hippocampus of AD brains [69].

#### 7.1.6. Oxidative Stress and Neuroinflammation Interplay

Many neurodegenerative diseases, including AD, Parkinson’s disease (PD), and amyotrophic lateral sclerosis (ALS), are associated with inflammatory responses characterized by the presence of pro-inflammatory cytokines, chemokines, and reactive gliosis [70]. Chronic oxidative stress has been connected with these neurodegenerative diseases and stimulates pro-inflammatory gene transcription in microglia and astrocytes, leading to various inflammatory reactions [71]. In the brain, microglia maintain homeostasis by responding to stress, trauma, or pathology via the engulfment of amyloid plaques in the case of AD. However, overstimulation of microglia and astrocytes due to ROS leads to cytotoxic effects and activates downstream pro-inflammatory signaling pathways, including NF-ĸB, TNF-α, IL-6, IFN-γ, IL-12p40, Cox-2, and IL-1β. These pathways are responsible for neuroinflammation, synaptic loss, and neuronal damage. The excessive production of pro-inflammatory cytokines further exacerbates the activation of both microglia and astrocytes, resulting in a condition known as gliosis, which promotes AD pathology [72]. Microglia not only express several variants of genes (*MS4A*, *CD2AP*, *BIN1*, *INPP5D*, and *SPI1*) involved in AD but also a rare variant encoding the triggering receptor expressed on myeloid cells 2 (TREM2) protein, which increases AD pathology five-fold. In addition, several studies prove that microglia deficient in the TREM2 receptor show strong metabolic consequences, including ATP depletion and increased oxidative stress markers [73,74].

Similarly, NADPH oxidase 2 (NOX2) specifically generates ROS in microglia, leading to damage-associated molecular patterns (DAMPs) or pathogen-associated molecular patterns (PAMPs) in the form of disease-associated microglia (DAM), which are crucial for inducing inflammation-associated neurodegeneration [75]. Furthermore, DAMs/PAMPs activate other microglial pattern recognition receptors (PRRs), called toll-like receptors (TLRs), which possess different domains: (i) an ectodomain for DAMs/PAMPs recognition; (ii) an embedded transmembrane domain; and (iii) the last cytoplasmic domain, the Toll/IL-1 receptor (TIR), for downstream signaling. The same study elaborates that the activation cascade of TLRs leads to Aβ aggregates and finally the generation of an inflammasome complex [76]. Multiple studies have demonstrated that extraneous and endogenous signals in the form of oxidative stress (NOX2) or Aβ oligomers activate TLR2 and TLR4 receptors, which further transmit signals via myeloid differentiation factor 88- (MyD88-) to stimulate NF-κB and generate inflammatory stress in the form of pro-inflammatory cytokines [77]. Similarly, the primary signal from NF-κB and downstream inflammatory cytokines, followed by ionic dyshomeostasis, mitochondrial ETC imbalance, and amyloid fibrils, can lead to activation of the NLRP3 (NLR family pyrin domain containing 3) inflammasome. Further, this NLRP3 inflammasome, along with ASC, migrates from the cytoplasm toward the mitochondria [78]. Several models provide evidence that mitochondrial dysfunction, ROS derived from NOX4, and Aβ can trigger NLRP3 activation. This activation, in turn, leads to NFTs as well as microglia-derived ASC specks generated by pyroptosis, hence resulting in synaptic loss [79,80].

## 8. Antioxidant Therapy

Although there are many therapies for AD, some are still in clinical trials, while others are available in the form of antioxidants, immunotherapeutics, and monoclonal antibodies specifically targeting Aβ or Ptau. However, the gap is largely filled only with symptomatic management. Since oxidative stress plays a significant role in the pathogenesis and progression of AD, considerable efforts have been made to use natural antioxidants to mitigate or decrease oxidative stress in the brain. Several studies using natural antioxidants, either in single or combination formulations, in cellular or animal models have significantly prevented and treated oxidative stress-induced neurodegenerative disorders [80]. Globally used antioxidants include carotenoids, polyphenols (flavonoids), vitamins, quinones, fatty acids, enzyme cofactors, polysaccharides, alkaloids, and amino acid derivatives, as well as hormones such as melatonin, which have been examined as supplementary treatments in neurodegenerative conditions (Table 1).

Polyphenols are natural extracts used in many studies involving the mouse hippocampal cell line HT-22 to evaluate the protective effects of various flavonoids, including resveratrol, epicatechin gallate, catechin, quercetin, and apigenin. The results show that they can protect neuronal tissue by enhancing the absorption of cystine, increasing the concentration of antioxidant enzymes, and consequently decreasing the ROS levels in the HT-22 cell line, which may benefit the management of neurological conditions [84]. Similarly, soy isoflavone and genistein have antioxidant properties confirmed in Aβ25–35-treated cultured hippocampal neurons, as well as in the d-galactose (DG)-induced C57BL/6J (B6) mouse model of aging. Higher concentrations of these compounds halt the rise of intracellular free calcium, ROS buildup, and caspase-3 activation [105,106]. Anthocyanins are natural water-soluble flavonoids found in fruits and vegetables that have a pronounced interaction with Keap1/Nrf2 signaling. By translocating Nrf2 into the nucleus, they result in the production of numerous detoxification and antioxidant enzymes such as nicotinamide adenine purine phosphate (NADPH), ureol reductase 1 (NQO1), glutathione S-transferase (GST), HO-1, glycine ligase (GLC), and catalase. These enzymes protect cells by eliminating ROS such as superoxide anions, hydrogen peroxide, and hydroxyl radicals [107]. These results prove that polyphenols significantly reduce oxidative stress and mitochondrial dysfunction and have been used to treat AD.

Next, the carotenoids that are most frequently described are lutein, lycopene, zeaxanthin, β-carotene, and α-carotene, which possess a strong ability to scavenge free radicals and protect against oxidative stress damage from tumors such as prostate and breast cancer [108]. One survey showed that three ingredients—lutein, lycopene, and zeaxanthin—were linked to a lower risk of death due to AD in a cohort of 6958 participants aged 50 years and older [109]. Various research studies have demonstrated that lycopene has multiple neuroprotective effects in both in vitro and in vivo models of AD by inhibiting oxidative stress and neuroinflammation. Specifically, transgenic mice bearing the P301L mutant tau protein were given a daily dosage of 5 mg/kg of LP for eight consecutive days, which successfully reversed elevations in serum malondialdehyde (MDA) and restored decreases in GSH activity. Also, this treatment regimen reduced the phosphorylation of tau protein at Thr231, Ser235, Ser262, and Ser396 in vivo [110]. This suggests that carotenoids may have anti-AD benefits by inhibiting tau phosphorylation, oxidation, and inflammation in neuronal tissue.

Furthermore, multivitamins (A, B, C, D, and E) are exogenous substances that protect neuronal tissue via their potent ability to scavenge ROS by donating electrons or chelating transition metal ions [111]. One qualitative, randomized, controlled, double-blind study evaluated the effects of daily administration of 2000 IU of vitamin E or 10 mg of selegiline, either alone or in combination, and found a significant reduction in AD symptoms in 341 participants [112]. In addition, the combined effect of vitamin C and D works together to protect cells from oxidative damage. Tappel suggested that fat-soluble vitamin E in its oxidized form is effectively reduced by water-soluble vitamin C, hence providing a synergistic effect [113]. Vitamin B is being investigated for its effects on variables such as alterations in phosphorylated tau, brain energy metabolism, oxidative stress, and cognitive function; however, numerous clinical trials are still in progress [114].

Coenzyme Q10 (CoQ10) functions as a lipid antioxidant and an electron carrier within the mitochondrial electron transport chain. Its presence and activity are essential for optimal mitochondrial performance and cellular energy production, thus helping to prevent mitochondrial dysfunction and the development of Aβ and tau pathologies in AD [91]. CoQ10 and vitamin E possess synergistic antioxidant behavior by effectively recycling the resulting vitamin E phenoxyl radical back into its physiologically active reduced form with the help of both fully reduced CoQH2 (ubiquinol) and semi-reduced CoQH (ubisemiquinone) [92]. In cell cultures as well as mouse models of AD, the administration of mitochondrial (MitoQ) enzymes was reported to improve memory retention, boost synaptic connections and neurite outgrowth, and decrease Aβ-induced oxidative stress [91].

Silibinin, an antioxidant flavonolignan commonly known as silybin and the primary active ingredient of silymarin, can increase the number of newly formed microglia, astrocytes, and neuronal precursor cells in the brain [93]. Various studies show that silibinin is a dual inhibitor of AChE and Aβ peptide aggregation. It also decreases neuronal damage induced by oxidative stress from Aβ1-42, suggesting a potential therapeutic approach for treating AD [94,115].

The curry spice curcumin has neuroprotective activity, including antioxidant, anti-inflammatory, and especially anti-protein aggregate effects [95]. Curcumin, at both low and high doses, dramatically reduced the levels of oxidized proteins, interleukin-1β, the astrocytic marker GFAP, insoluble β-amyloid (Aβ), soluble Aβ, and plaque load by 43–50% in a transgenic mouse model [116]. Furthermore, in vitro, incubation of PC12 cells with Indian spicy curcumin mitigated the elevation of intracellular calcium levels and tau hyperphosphorylation, while also enhancing the repair of damaged DNA and antioxidant enzyme levels induced by Aβ25–35 [117].

Alpha-lipoic acid (α-LA), a naturally occurring antioxidant found in the body and in foodstuffs, has been seen as a potential therapeutic or preventive measure for neurodegenerative disorders when taken as a dietary supplement [96]. α-LA shows multidimensional effects, including activation of choline acetyltransferase (ChAT) for acetylcholine (ACh) metabolism and deactivation of the GSK-3β pathway for tau phosphorylation via stimulation of protein kinase C in vitro. Similarly, α-LA can chelate numerous metal ions (Hg^2+^, Fe^3+^, Zn^2+^, Pb^2+^, and Cu^2+^), and incubation of neuronal tissue with α-LA causes a significant downregulation of both active iron reserves and iron absorption inside neurons. In addition, α-LA reverses age-linked cognitive decline, such as mitochondrial dysfunction, either by suppressing cytoplasmic glutaredoxin-1, which maintains the integrity of mitochondria and voltage-dependent anion-selective channel (VDAC) redox levels, or by impairing enzyme cofactors such as pyruvate dehydrogenase (PDH) and alpha-ketoglutarate dehydrogenase (KGDH) [118].

Brain glutathione (GSH) is a major non-enzymatic antioxidant that plays a vital role in preserving the redox state of the brain and shielding its cells from oxidative stress via ROS scavenging. It also acts as a substrate for thiol oxidoreductase enzymes, as well as its oxidized form, glutathione disulfide (GSSG) [97]. One study revealed that the GSH/GSSG ratio in the brains of an AD transgenic mouse declined as the disease advanced, occurring before amyloid plaque formation. This was followed by a steady increase in GSSG levels and a corresponding decline in the GSH/GSSG ratio [119].

Polysaccharides such as Astragalus membranaceus (APS), a perennial herb, have been demonstrated to have neuroprotective, anti-inflammatory, and antioxidant properties. The study reveals that an aqueous extract of APS can reverse memory impairment by enhancing the number of M-cholinergic receptors in the cortex, hippocampus, and striatum of an aged rat model. Additionally, it prevents axon and synapse loss in the cortex and hippocampus of Aβ-induced cognitive deficit mice [98]. Furthermore, APS reduces chronic brain inflammation by inhibiting the hyperactivity of microglia and astrocytes, which are commonly activated near brain plaques in AD patients [120].

Nicotine is an antioxidant that belongs to the class of alkaloids and is mainly found in tobacco leaves. Interestingly, low doses of nicotine administered to AD patients enhance attention and information processing capabilities as well as improve memory impairment [99]. The investigators suggest that nicotine may reduce Aβ accumulation by increasing cholinergic transport or targeting the secondary structure of Aβ. In another study, nicotine was found to prevent NF-kB and c-Myc activation via MAPKs pathway inhibition in London mutant V717I mice. Consequently, there was a down-regulation of inducible NOS activity and NO generation [100]. Furthermore, compared to vitamin C, nicotine has a greater ability to scavenge hydroxyl and superoxide radicals, suggesting it may be a potential candidate for AD treatment [121].

Palmatine (PA), an isoquinoline alkaloid, has significant biological effects, including anti-acetylcholinesterase (AChE), anti-inflammatory, antioxidant, and antidepressant properties. The same study proves that PA exhibits potent and concentration-dependent antioxidant effects when combined with trolox and ascorbic acid [101]. Another study demonstrated that PA may have anti-AD properties through cholinesterase inhibition, reduction in Aβ aggregation, low production of reactive oxygen species (ROS), and attenuation of oxidative damage [122].

N-methyl-(2S,4R)-trans-4-hydroxy-L-proline (NMP), derived from Sideroxylon obtusifolium, has anti-inflammatory and antioxidant properties and is used as a folk medicine in Brazil. NMP significantly reduced oxidative stress, as indicated by the increased expression levels of NRF2/HO-1, and mitigated synaptic failure by enhancing the levels of pre-synaptic and post-synaptic proteins in the cortices and hippocampi of Aβ1–42-injected mice, thereby improving cognitive function [103].

N-acetyl-L-cysteine (NAC) is a small molecule with a thiol group that has antioxidant qualities and easily enters intracellular spaces as well as the blood–brain barrier. Firstly, NAC serves as a cysteine donor, the substrate that limits the rate of glutathione synthesis. Secondly, NAC can function by reacting directly with ROS through its reducing thiol group. Thirdly, it has been demonstrated that in cultured neural cells, NAC can prevent apoptosis and is less toxic as an antioxidant compared to cysteine itself in the central nervous system [104].

## 9. Targeting Aβ with Various Approaches

Although Aβ therapies are still under clinical trials, certain drug classes, such as cholinesterase inhibitors (galantamine, rivastigmine, donepezil, and tacrine), are used to treat mild-to-moderate or late-stage AD in patients. These medications work by blocking the cholinesterase enzyme, thereby increasing acetylcholine concentration. Another class includes memantine, an NMDA receptor antagonist that improves the symptoms of Alzheimer’s disease by inhibiting glutamate activity [123]. In addition, some monoclonal antibodies and small molecules directly interact with the Aβ peptide or its aggregates and fall into four categories: (1) reduce the production of Aβ; (2) accelerate Aβ breakdown and remove Aβ aggregates; (3) neutralize soluble Aβ monomers or their toxic effect; and (4) directly prevent Aβ aggregation [124,125,126]. Several monoclonal antibodies were stopped due to no effect or deleterious effects. Bapineuzumab (Elan/Pfizer Inc./Johnson & Johnson) and Solanezumab (Eli Lilly and Company) were discontinued in phase 3 clinical trials because they did not meet primary goals. Similarly, the vaccine AN1792, the first anti-amyloid beta (anti-Aβ) vaccine developed by Elan, was stopped in phase 2 due to aseptic meningoencephalitis. On the other hand, patients who had an antibody response to the vaccine showed less cognitive decline in follow-up research, indicating that Aβ immunotherapy might have clinically meaningful effects. The same study reported that the monoclonal antibody BAN2401, which is a humanized version of mAb158, exhibits selectivity for protofibrils that is at least 1000 times higher compared to Aβ monomers. This allows it to target the hazardous species of the peptide [127]. Aducanumab is a fully humanized IgG1 monoclonal antibody that works by dissolving β-amyloid clumps into smaller oligopeptides, or amino acids. A monthly infusion of aducanumab for a year reduced brain Aβ plaques in a time- and dose-dependent manner, as observed in double-blind, randomized placebo-controlled phase 1b research (PRIME) and phase 3 studies (ENGAGE and EMERGE) [128]. Unfortunately, two types of adverse effects were observed in clinical trials and confirmed via magnetic resonance imaging (MRI): ARIA-H (hemosiderin deposition in brain parenchyma) and ARIA-E (sulcal effusion), which created some controversy regarding the safety and approval of aducanumab [129]. Concerning monoclonal antibody use in AD, the FDA has approved aducanumab and lecanemab out of 38 other antibody-based medications, that were terminated because of harmful side effects or a lack of efficacy (Table 2).

### 9.1. Targeting Aβ at Various Prospectives

#### 9.1.1. Aβ and BACE1 Inhibitors

As BACE1 is the rate-limiting β-secretase for Aβ production, several researchers have proposed the discovery of potent BACE1 inhibitors in numerous therapeutic trials, as blocking BACE1 activity is considered a crucial strategy for stopping the amyloid cascade. LY2886721 was the first anti-BACE1 agent that, in phase 1 studies, showed reduced levels of Aβ40 and Aβ42 in cerebrospinal fluid (CSF), as well as increased sAPPα levels. However, it showed elevated liver function test (LFT) values in phase 2 clinical trials [135]. Other BACE1 inhibitors include lanabecestat (phase 2/3), atabecestat (phase 2/3), and elenbecestat (phase 3), all of which effectively reduce Aβ in the later stages of AD [136,137]. As BACE1 has a large active site with structural similarities to aspartyl proteases such as pepsin, renin, and BACE2, it is difficult to occupy by the above small molecules BACE1 inhibitors as well as its selective inhibition.

#### 9.1.2. Aβ and γ-Secretase Inhibitors

Inhibitors of gamma-secretase (GSIs), such as avagacestat (phase 2) and semagacestat (phase 3), have demonstrated significant effects on lowering Aβ levels in animal models and are potential therapeutic approaches for AD. However, both drugs were terminated due to gastrointestinal and dermatological adverse effects, particularly disturbances in Notch signaling [138]. On the contrary, the same study demonstrated that γ-secretase modulators are alternatives to GSIs for reducing Aβ levels. They found that some non-steroidal anti-inflammatory drugs (NSAIDs), such as Sulindac sulfide, Ibuprofen, and Indomethacin, might modulate the γ-secretase enzyme to reduce Aβ42. Similarly, Itanapraced (CHF5074), a derivative of NSAID developed by a Chinese pharmaceutical company, underwent clinical testing. However, in a phase 1 study, it did not impact CSF Aβ42 levels [139]. Additionally, non-NSAIDs (E2012 and E2212) performed their anti-Aβ effect up to ~50% in human phase 1 trials but were associated with unacceptable cholesterol metabolism, leading to lenticular opacity [140].

## 10. Limitations, Low Specificity, and Future Directions

Optimization of drugs for treating any disease requires the right dose, the right patient, the right route, and the right time. However, in AD, we still lack the specificity and selectivity of drugs in clinical trials. In this review, we highlight the detailed pathophysiology of AD, including the amyloid aggregation cascade, tau phosphorylation, oxidative stress-induced mitochondrial dysfunction, neuroinflammation, and neurodegeneration. In this narrative review, we have thoroughly outlined the potential of carotenoids, vitamins, flavonoids, alkaloids, and mitochondria-targeted antioxidants, as well as immunotherapeutics, against the onset and progression of AD. Although natural antioxidants have shown intriguing effects in disease models of AD, it does not appear that we can currently optimize them for use as human therapeutics. One possible reason for the lower effectiveness of antioxidants is the decreased transmembrane potential of injured mitochondria, which may accumulate fewer antioxidants than functional mitochondria [141]. Another problem concerning the duration of antioxidant therapy is that most studies administer antioxidants for a specific time frame; hence, the effectiveness of long-term care is still not well supported by data [142]. In addition, low specificity and selectivity present several limitations, which are as follows: Firstly, γ-secretase and BACE1 inhibitors have numerous substrates or target incorrect substrates, leading to toxicity due to selective inhibition of Notch-1 cleavage or BACE2, a close homolog of BACE1 with neuroprotective effects [143]. Secondly, the lack of accurate animal models is a limitation. Transgenic mouse models containing *APP* gene mutations (e.g., APP SweDI and TgCRND8 mice), *PSEN1* gene mutations (e.g., PS1A246E mice), or their combinations (e.g., APPSwe/PSEN1dE9) cannot accurately depict the sporadic form of AD, which typically occurs at an age greater than 60 years [144]. Similarly, human tau and APP transgenic mouse models, which include APP/PS1/rTg21221 and 3xTg-AD, as well as AD knock-in/out mouse models that precisely target specific loci, such as APPNL-G-F knock-in, all show an early onset of the disease and resemble familial AD [145]. Thirdly, the diagnosis of AD by various techniques is often too late, and the trial population shows severe disease progression by the time AD therapy is initiated. Although CSF Aβ levels can be detected up to 25 years before symptom onset via PET imaging, this is often too late for effective intervention [146].

Since numerous studies have indicated that mitochondrial dynamics and mitophagy play a crucial role in the development of neurodegeneration, these processes may be attractive targets for therapeutic intervention [147]. Furthermore, when planning the dosage and administration schedule, consideration should be given to the metabolic changes that typically accompany aging. This depends on several factors, including the prescribed treatment as well as food and supplement consumption, to avoid excessive accumulation of antioxidants [141]. Additionally, recent developments in nanotechnology and nanomedicine have led to the creation of encapsulated antioxidants such as liposomes, niosomes, and transferosomes to overcome the limitations associated with antioxidants. These include instability in aqueous mediums, low efficacy, lack of specific targeting activities, inability to cross the blood–brain barrier (BBB), and potential side effects at high doses. Similarly, many approaches are currently being investigated, including nanoparticle therapy, lipid-based nanoparticles, and antibody-coated nanoparticles, to enhance the biopharmaceutical properties of antioxidants and immunotherapeutics targeting promising proteins for AD [148,149]. When selecting monoclonal antibodies and vaccines for AD clinical trials, several factors must be carefully taken into account, including the patient population, the mechanism of action, the choice of epitope, and the antibody subclass and charge [147]. We assure you that research in the fields of biogenetics and bioengineering (biosensors) may open up new avenues for the treatment and eventual cure of AD. For example, potential applications include a selective copper detector probe that can identify Aβ peptide-copper aggregates and reduce their aggregation size, a simple sandwich electrochemical immunosensor for the rapid identification of tau protein, and AChE-based biosensors for the quick and accurate detection of medications that can reversibly inhibit AChE activity. These biosensors hold significant promise as alternative techniques for the rapid, accurate, and sensitive detection of early biomarkers for AD biomarkers [150,151,152]. Human-based models in AD include three-dimensional (3D) brain organoids, xenografted microglia produced from human induced pluripotent stem cells (iPSCs), human postmortem brain tissue, and microfluidic devices, all of which hold great promise for representing neural diseases (NDs) in a complex, tissue-like environment. Total and single-cell multi-omics research utilizes the combined knowledge from transcriptomics (total RNA sequencing), metabolomics (a small biological molecule having a weight less than 1.5 kDa), and proteomics to figure out the cellular characteristics and roles of the diverse populations in organoids. This approach helps to validate neurodegenerative models and explores directions for further study to gain a deeper understanding of AD [153]. With the limited effectiveness of amyloid-β-targeting treatments for AD, attention has shifted to tau protein expression, post-translational changes, aggregation, and clearance, which are now being tested on human subjects. Antisense oligonucleotides and immunotherapies that can interact intracellularly and/or extracellularly with tau proteins have demonstrated encouraging outcomes in human trials [154]. In addition, other future perspectives that can be favorable for Aβ-targeted drug development include improving amyloid-beta combination therapies and mechanistic-based immunotherapies, such as BACE1 inhibitors and γ-secretase inhibitors, for the early stages of the amyloid cascade or protofibrils. Additionally, optimizing trial populations, discovering new biomarkers to investigate the early stages of sporadic AD, utilizing more sensitive cognitive composite scales like the AD Composite Score, improving the safety profiles of Aβ immunotherapy drugs, and developing patient-based research models such as brain organoids and xenotransplantation are important.

## 11. Conclusions

Since AD is the leading cause of various neurodegenerative disorders, it is characterized by Aβ burden, oxidative stress, neuroinflammation, and ultimately synaptic and memory impairments. The Aβ cascade and oxidative stress in AD pathogenesis are bidirectional and interdependent processes that aggravate each other. In this vicious cycle, oxidative stress is induced by Aβ, leading to increased Aβ synthesis and aggregation, which in turn results in further oxidative damage, creating a continuous positive feedback loop. Failure to develop accurate human translational models, along with low efficacy and specificity and the lack of a therapeutic window, are reasons for unsuccessful Aβ targeting. Consequently, Aβ aggregates, phosphorylated tau, their genetic profiles, and ROS are viewed as promising targets for modifying AD-related pathological consequences. Using a combination of natural antioxidants and monoclonal antibodies, guided by biosensors for the specific identification of AD biomarkers and related patients with preclinical signs and symptoms, may help slow down or prevent the disease. We assure that these solutions may help to perform novel research, especially targeting Aβ-based signaling pathways, with more sophistication and accuracy.

## Figures and Tables

**Table 1:** Natural antioxidants target various ROS-induced signaling pathways in AD.

Active Ingredient	Mechanism of Action (MOA)	Outcome Goals	Reference
Resveratrol	PI3K/Akt/Nrf2 pathway	Reduced ROS, elevated antioxidant enzymes GST and SOD, and activated PI3K, Akt, HO-1, and Nrf2 pathways.	[81]
Epicatechin gallate Green tea	Iron-chelating, activating ERK, Akt/PKB, PI3K, and PKC pathways	Reduces both amyloid plaques and ROS, promotes the production of SAPPα	[82]
Quercetin	Nrf2/ARE pathway reduces the level of MDA in an animal model	Increase antioxidant enzymes SOD, HO-1, Catalase, and scavenging ROS	[83]
Apigenin	C2–C3 double bond on the C ring reduces hydrogen peroxide and ROS ERK1/2/CREB/BDNF pathways	Reduce oxidative stress, Synaptic improvement	[84]
Anthocyanins	Reduce glutamate-induced AMPK activation, ros, and pro-inflammatory cytokines	Glutamate-induced neurotoxicity, neuroinflammation	[85]
Melatonin	SIRT1/Nrf2 pathway, anti-amyloidogenic properties	oxidative stress damage, apoptotic neurodegeneration	[86]
Lycopene	Nrf2/ARE pathway, reducing the phosphorylation of the tau protein	Increased antioxidant enzymes as well as anti-AD benefits	[87]
Osmotin	TLR4/NFκB signaling and SREBP2 via the AdipoR1/AMPK/SIRT1 pathway	Neuroinflammation, improved pre- and post-synaptic dysfunction	[88]
Vitamin C	Preserves the mitochondria’s cellular membrane integrity, scavenging ROS	Antioxidant potential in AD, change in cognitive function	[89]
Vitamin E	Tyrosine hydroxylase and Nurr1 expression, scavenging ROS	Antioxidant, neuroprotective, anti-inflammatory, and cholesterol-lowering properties	[90]
Coenzyme Q10	As a cofactor for electron carriers in ETC, inhibits lipid peroxidation, recycles vitamin E phenoxyl radical	Bioenergetic modulator, antioxidant, boost synaptic connection and neurite outgrowth	[91,92]
Silibinin	Aβ and AChE inhibitors, lowering the production of H_2_O_2_	Could increase the number of neuronal precursor cells in the brain, reduce oxidative stress	[93,94]
Curcumin	Increased GSH level, reduced insoluble Aβ, soluble Aβ, tau hyperphosphorylation, and intracellular calcium levels	Anti-protein aggregates, antioxidant, epigenetic potential in AD	[95]
Alpha-lipoic acid	ChAT enzyme activation, stimulation of phosphokinase C, metal chelation	As a dietary supplement for AD, reduces age-linked cognitive decline	[96]
Glutathione	substrate for thiol oxidoreductase and oxidized glutathione disulfide, reduces hydrogen peroxide, peroxynitrite, and lipid hydroperoxides	Maintain the redox state of the brain	[97]
Astragalus membranaceus	Increase the number of M-cholinergic receptors in the brain, inhibit reactive gliosis	Alleviating AD-related metabolic pathologies	[98]
Nicotine	Cholinergic secretagogue, down-regulation of inducible NOS, inhibition of NF-kB and c-Myc pathways, scavenging of hydroxyl and superoxide radicals	Enhance retention, and learning abilities in AD patients, antioxidant	[99,100]
Palmatine	Anti-AChE, AMPK/mTOR autophagy signaling system	Anti-depressant, anti-inflammatory, antioxidant	[101,102]
N-methyl-(2S, 4R)-Trans-4-hydroxy-L-proline	Elevate expression levels of NRF2/HO-1 and pre-synaptic and post-synaptic proteins	Anti-inflammatory, antioxidant, improving cognitive improvement	[103]
N-acetyl-L-cysteine	Increased GSH level, cysteine donor, cross BBB	Cease apoptosis in CNS, improved dementia rating scale	[104]

**Table 2:** Immunotherapy containing monoclonal antibodies and vaccines for targeting Aβ.

Active Ingredient	Mechanism of Action (MOA)	Outcome Goals	Reference
Bapineuzumab	Aβ in a monomeric helical conformation at the N-terminus	An early biomarker of AD	[130]
Solanezumab	A humanized monoclonal IgG1 antibody that decreases Aβ-induced synaptic toxicity by targeting the Aβ peptide’s mid-domain	elevated levels of plasma Aβ and lowered levels of CSF Aβ40, synaptic re-modelling in phase 2/3 clinical trials	[131]
AN1792	Amyloid-beta (anti-Aβ) vaccine	Senile plaque disruption	[132]
Gantenerumab	Human IgG1 antibody that binds to a conformational epitope on Aβ fibrils to stimulate phagocytosis by attracting microglia	Reduced plaque load	[133]
BAN2401	A monoclonal antibody with a humanized version of mAb158, selectivity for protofibrils	Aβ immunotherapy in early AD	[127]
Lecanemab	A humanized IgG1 binds to large soluble Aβ protofibrils (Approved by the FDA)	Reduced brain amyloid and improved cognitive decline in phase 3 clinical trials	[134]
Aducanumab	Fully humanized IgG1 mAb target conformational epitope present on the N-terminus of Aβ, dissolving β-amyloid clumps into smaller oligopeptides (Approved by FDA in June 2021)	Significantly improved cognitive deficits with high dose of intravenous infusion in randomized phase 1b and phase 3 clinical trials	[128,129]

## Data Availability

Not applicable.
